# Acupuncture–Moxibustion Combined with Locomotor Training Enhances Postoperative Recovery in Canine Cervical Intervertebral Disc Herniation: A Pilot Study

**DOI:** 10.3390/ani15203038

**Published:** 2025-10-20

**Authors:** Tianyu Wang, Dongchun Jin, Wen Cui, Jincheng Bai, Han Zhang, Jiayi Wang, Inseong Jeong, Xinmei Jin, Namsoo Kim, Zhenglin Piao

**Affiliations:** 1 Department of Veterinary Medicine, College of Agriculture, Yanbian University, Yanji 133002, China; tianyu_vet@126.com (T.W.); jindongchun@ybu.edu.cn (D.J.); zhanyyhy@163.com (H.Z.); wjy80555088@sina.com (J.W.); 0000008766@ybu.edu.cn (X.J.); 2 Yanhong Branch, Ringpai Pet Hospital, Yanji 133099, China; cuiwen@ringpai.com; 3 Heping Branch, Ringpai Pet Hospital, Shenyang 110002, China; baijincheng@ringpai.com; 4 Royal Animal Medical Center, Seoul 02117, Republic of Korea; jung45457@hanmail.net; 5 Department of Veterinary Surgery, College of Veterinary Medicine, Jeonbuk National University, Iksan 54596, Republic of Korea

**Keywords:** Canine cervical IVDH, Acupuncture–Moxibustion, Locomotor training, Rehabilitation, Ventral slot decompression, electroacupuncture, Rusbridge grade, Propensity Score Matching, Postoperative recovery

## Abstract

This study aimed to evaluate the effects of acupuncture–moxibustion (AM) combined with Locomotor Training (LT) on functional recovery after surgery for Cervical Intervertebral Disc Herniation (IVDH) in dogs. Seventy-nine dogs undergoing ventral slot decompression (VSD) between 2022 and 2025 were enrolled and assigned to either the AM plus LT group (ALRG group) or the LT-only group (LRG group) based on postoperative rehabilitation protocols. To control for bias, post hoc analysis used propensity score matching (PSM) stratified by Rusbridge Grade. Primary outcomes included time to standing and walking recovery, along with Olby scores at five postoperative time points. Secondary endpoints included postoperative analgesic usage rate and duration, as well as long-term prognosis at 6–8 months postoperatively. After PSM, each group comprised 20 dogs. Compared with the LRG group, the ALRG group demonstrated shorter time to standing and walking recovery, better Olby score improvement, and significantly reduced opioid usage duration. Long-term follow-up revealed a higher complete success rate in the ALRG group. All trends were more pronounced in severe cases classified as Rusbridge grades 3 and 4. This study demonstrates the clinical feasibility of combining AM with LT rehabilitation for postoperative recovery in dogs with cervical IVDH. It provided new evidence for optimizing postoperative rehabilitation protocols and supports future large-scale prospective studies.

## Simple Summary

Canine Cervical Intervertebral Disc Herniation is a common neurological disorder in clinical practice. While ventral slot decompression effectively relieves spinal cord compression, postoperative functional recovery remains highly dependent on rehabilitation therapy. Existing studies indicate that multimodal exercise training or combined adjunctive therapies demonstrate certain efficacy, yet their effectiveness remains limited in cases with severe neurological deficits. This study aimed to evaluate the role of acupuncture–moxibustion combined with Locomotor Training versus exercise training alone in the postoperative rehabilitation of dogs with cervical intervertebral disc herniation. By analyzing indicators such as time to regain standing and walking, Olby scores at five time points, analgesic medication usage, and long-term prognosis, it was found that acupuncture–moxibustion combined with Locomotor Training promoted motor function recovery and improved long-term neurological outcomes.

## 1. Introduction

Canine intervertebral disc herniation (IVDH) is a disease characterized by the dorsal protrusion of a degenerated disc into the vertebral canal, compressing the spinal cord and primarily causing movement disorders or cervical pain. It is the most common cause of nerve root characteristics in dogs [1]. IVDH is classified into Hansen types I and II [2]. Small dogs, especially breeds with chondrodysplasia, have a higher probability of developing Hansen type I IVDH [3], and it frequently affects the cervical spine [4]. Hansen type II IVDH, on the other hand, often occurs in older, large dogs aged 7 years and older [2,5]. Overall, the majority of IVDH cases occur in the thoracolumbar region (approximately 65–85% of cases) [6,7], while cervical IVDH accounts for approximately 14–25% of all cases [4,8,9]. MRI accurately diagnoses both Hansen type I and Hansen type II IVDH and is considered the gold standard for diagnosing canine IVDH [10,11].

Currently, the treatment of canine cervical IVDH is categorized into conservative therapy and surgical intervention, with conservative therapy usually only applied to dogs with mild clinical signs and mild spinal cord compression [12,13], and with a high recurrence rate [13,14]. If the dog does not respond well to conservative treatment or if the spinal cord compression severely affects the dog’s quality of life, surgical treatment is required [15]. Surgical approaches include ventral slot decompression (VSD), VSD with fixation, the modified slanted slot, dorsal laminectomy, and cervical hemi-laminectomy, with VSD considered the preferred procedure because of its direct decompression effect [16,17,18,19].

Despite the efficacy of VSD, the prognosis is related to the preoperative neurological status, timing of surgery, and risk of complications, and some dogs continue to have a poor prognosis or develop severe or mild complications and adverse effects [9,19,20,21]. The intervention of appropriate rehabilitation therapy may help in restoring neurological function [22,23,24,25]. Rehabilitation is now a key component of postoperative rehabilitation in canine IVDD.

Locomotor training (LT), which in some cases is considered a form of physical therapy, is the most researched area of veterinary rehabilitation and has become an important component in the rehabilitation of neurological disorders such as canine cervical IVDH. Existing clinical practice has shown that exercise training combined with therapies such as manipulative therapy, electrotherapy, and infrared therapy is effective in postoperative cases of IVDH, but its therapeutic efficacy is still deficient in dogs with severe neurological dysfunction such as quadriplegia or hypoventilation [22,23,24,25,26,27].

Acupuncture–moxibustion (AM), as a traditional medical therapy that originated in ancient China, has had a proven and reliable efficacy for thousands of years. Modern scientific studies have gradually revealed the mechanism of action of AM. AM usually includes two therapies, acupuncture and moxibustion, and acupuncture is subdivided into manual acupuncture (MA), electroacupuncture (EA), and blood acupuncture. Currently, AM has also been used in the conservative treatment of canine intervertebral disc disease [28,29,30,31,32]. More notably, AM not only promotes the recovery of neuromuscular function [33,34,35] but also has multiple therapeutic effects such as analgesia and anti-inflammation, the elimination of nerve root edema, and the promotion of tissue repair [36,37,38]. It may have strong advantages in the postoperative rehabilitation of canine cervical IVDH.

This study, through retrospective propensity score matching analysis, introduced AM into postoperative rehabilitation for canine cervical IVDH for the first time within the existing literature framework. It aimed to compare the feasibility and efficacy of AM combined with LT for functional recovery after canine cervical IVDH surgery, thereby providing evidence-based support for optimizing postoperative rehabilitation strategies.

## 2. Materials and Methods

### 2.1. Study Design

This retrospective cohort study retrieved the electronic medical record database of Heping Branch, Ringpai Pet Hospital to screen dogs diagnosed with cervical IVDH between 2022 and 2024. Informed consent was obtained from the owner prior to any diagnosis and treatment of each dog; the study protocol was approved by the Experimental Animal Welfare Ethics Committee of Yanbian University (No.: YD20250722001).

Inclusion criteria included dogs that received a diagnosis by MRI and underwent surgical treatment and postoperative rehabilitation after diagnosis. The dogs were divided into two groups according to the postoperative rehabilitation procedures chosen by the owners.

The AM combined with LT group (ALRG group) were dogs that underwent postoperative rehabilitation through AM combined with LT, and the LT rehabilitation alone group (LRG group) were dogs that underwent postoperative rehabilitation through LT only.

Exclusion criteria included dogs with a combination of severe systemic diseases, multisegmental IVDH, and other neurologic diseases. Dogs with minimal neurologic impairment were treated conservatively and were not included in the study. Cases that died within 3 days postoperatively were also excluded because early deaths were considered primarily attributable to iatrogenic spinal cord injuries or major intraoperative hemorrhage and were not relevant to the assessment of the efficacy of subsequent rehabilitation therapy.

Information extracted from the medical records included gender, age at admission, breed, weight, clinical history, anatomical localization of the IVDH, MRI, and procedures for surgery and rehabilitation. The preoperative version of the modified grading system by Rusbridge et al. [17] was used, as shown in Table 1.

The primary efficacy endpoints were the postoperative time to standing recovery (defined as the shortest time required to stand without assistance) and the time to walking recovery (defined as the shortest time required to walk more than ten steps without assistance). Additionally, Olby scores [39] based on neurological examination and observational gait analysis were assessed at specific postoperative time points (0d, 7 d, 14 d, 30 d, and 60 d) under identical environmental conditions. All images were recorded using a Canon EOS 2000D camera. To minimize assessment bias, all video recordings were independently evaluated by one veterinary rehabilitation therapists who were blinded to the group allocations.

Secondary efficacy endpoints included postoperative analgesic usage rates and duration of medication during hospitalization to assess differences in drug dependency between groups. Additionally, all dogs underwent follow-up evaluations 6 to 8 months postoperatively using a self-designed structured questionnaire completed by owners to assess long-term prognosis after neurological stabilization. The questionnaire covered four core functional domains: motor function, pain manifestation, urinary status, and medical dependence. Motor function was assessed on a 4-point scale ranging from normal (4 points) to unable to walk (1 point); pain frequency from absent (0 points) to frequent (3 points); urinary function from normal (0 points) to persistent abnormality (2 points); and medication dependency from no medication required (0 points) to daily medication (2 points). Based on the questionnaire results, overall outcomes were classified as complete success (motor function = 4 with all other domains = 0), partial success (motor function = 3 with all other domains ≤ 1), or minimal improvement (cases not meeting these criteria).

### 2.2. Diagnostic Imaging

All dogs received a uniform anesthesia regimen, starting with the induction of anesthesia by the slow intravenous injection of propofol 4–6 mg/kg, followed by the use of an anesthesia machine (Gavet-800, Beijing Gavet Medical Technology Co., Ltd., Beijing, China) to maintain anesthesia, and then anesthesia was maintained by the inhalation of isoflurane using an anesthesia machine (Gavet-800, Beijing Gavet Medical Technology Co., Ltd., China), while the inhalation of oxygen was used to maintain respiration, and vital signs were monitored.

**Figure 1:**
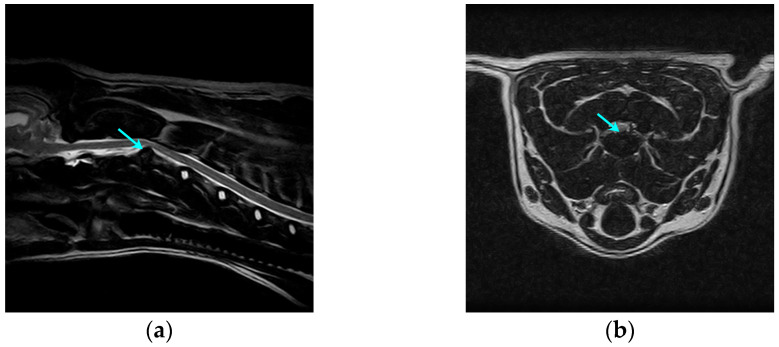
Preoperative MRI images of a dog with cervical IVDH showing a C2-C3 disc herniation (arrows). (**a**) Left sagittal plane; (**b**) cross-section.

MRI was performed using a 0.35 T magnetic resonance imaging device (Spike Technology Development Co., Ltd., Beijing, China). The MRI protocols included T1WI, T2WI, and FS-T2WI, and left-sided sagittal and cross-sectional images of the C1-C7 segments were acquired (Figure 1), with an imaging layer thickness of 3 mm. The results were all interpreted by one senior veterinarian in the imaging department. The presence of an abnormal disc material signal with compression of the spinal cord or nerve roots was used as a diagnostic criterion.

### 2.3. Perioperative Protocol

All dogs were preoperatively infused with 0.9% saline (10 mL/kg/hour) through an intravenous catheter, and the amount used was increased as appropriate for dogs that were dehydrated or hypovolemic. Ceftriaxone (30 mg/kg, bid) was administered intravenously to prevent infection and dexamethasone (1 mg/kg) to reduce spinal cord edema and inflammation. An intraoperative Constant-Rate Infusion (CRI) of Butorphanol (loading dose, 0.1 mg/kg; maintenance dose, 0.2–0.3 mg/kg, q1h) was administered to reduce pain.

After using the same anesthetic protocol as for the MRI examination, the VSD technique described by Shamir et al. [40] was administered for spinal cord decompression, and all procedures were performed by or under the supervision of a neurosurgical veterinarian.

During the first 72 h postoperatively, dogs were maintained on strict rest in the intensive care unit with close monitoring as well as fluid therapy and nutritional support. Concurrent medications included intravenous ceftriaxone (30 mg/kg, BID) and cimetidine (10 mg/kg, TID). Analgesia consisted of subcutaneous NSAIDs: meloxicam (0.2 mg/kg, SC/IV, QD) and carprofen (2.2 mg/kg, SC, BID). Opioids were administered based on the individual animal’s pain status, specifically butorphanol (0.05–0.1 mg/kg, IV, Q6–8H). For neuropathic or refractory pain, calcium channel modulators were supplemented, including gabapentin (10–20 mg/kg, PO, Q8–12H) and pregabalin (2 mg/kg, PO, Q8–12H).

### 2.4. Rehabilitation Intervention Protocol

#### 2.4.1. ALRG Group

The ALRG group started AM at 24–48 h postoperatively, and all dogs were treated with a uniform AM programme, which was developed based on Traditional Chinese Veterinary Medicine (TCVM) and related mechanism studies in recent years. The acupoint locations were determined with reference to *Canine Acupuncture Points and Atlas* [41,42] (Table A1). Three types of AM therapies were included: electroacupuncture, manual needling, and moxibustion; electroacupuncture points included Jing-jiaji (cervical jiaji) (2–3 pairs adjacent to the diseased segment), BL-11 (Da zhu), LU-7 (Lie que), LI-4 (He gu), KID-3 (Tai xi), BL-20 (Pi shu), BL-23 (Shen shu), BL-18 (Gan shu), and ST-36 (Zu san li), and the acupoints were shaved and sterilized topically. After using sterile acupuncture needles (0.25 mm × 13 mm, Beijing Zhongyan Taihe Medical Instruments Co., Ltd., Beijing, China) to allow their entry, a pair of acupoints on the same side of the body was connected with electrodes. The EA device (Indeer Medical Information System Co., Ltd., Shenyang, China) was set to stimulate the acupoints for 10 min with continuous waves at 40–80 Hz, followed by 10 min of 2/40–80 Hz sparse-dense wave stimulation, and then another 10 min of 2/40–80 Hz sparse wave stimulation. The current intensity was set to cause a slight tremor of the local muscles (0.5–1.2 mA).

Two points, GB-20 (Feng chi) and LIV-3 (Tai chong), were selected for MA, and after the needle was inserted and reacted with qi, a lifting and inserting twisting maneuver was performed every 3–5 min, and the needle was left in place for 15–25 min.

In addition, moxibustion was applied to ST-36, using moxa sticks (10 mm × 100 mm, Beijing Zhongyan Taihe Medical Instruments Co., Ltd., Beijing, China), which were burned until red-hot, and then a circular motion of 2 cm in diameter (1 circle every 2 s) was applied 3–5 cm from the skin, controlling the skin temperature at 40 ± 2 °C for a period of 15–25 min. The skin temperature was controlled at 40 ± 2 °C for 10–15 min.

The AM procedure was operated by a veterinary acupuncturist throughout the procedure, and mucosal coloration, heart rate, respiratory rate, blood pressure, muscle bundle tremor, and spinal nociceptive sensitization were closely monitored during the treatment. Initially every 3 days, it was gradually reduced to once every 7 days as the dog progressed through the healing process. The treatment period ranged from 21 to 60 days.

The LT procedure was identical to that of the LRG group, but it was necessary to ensure that the underwater treadmill (UWTM) training and AM were separated by more than 48 h, and all other LT protocols were performed 60 min after the end of AM, and the LT protocols were specified as follows.

#### 2.4.2. LRG Group

Massage was initiated 24 h post-surgery using bilateral symmetrical techniques, focusing on the neck and scapular muscle groups. On days 1–3 post-surgery, stroking was applied combined with cold compresses for 10 min per session, three times daily. Starting on day 4, the technique was transitioned to petrissage: gradual pressure was applied using the thumb and four fingers to grasp the muscle, maintaining muscle tension with circular compression. Each session was extended to 15 min. Pain response was continuously assessed throughout to adjust pressure intensity.

After 24–48 h postoperatively, Passive Range of Motion (PROM) was performed on each joint of the limbs by slowly flexing the dog’s joints to a painless but moderately resistive position, holding it for 10–30 s, and then slowly extending it to the same position, and repeating this 10–15 times a day to ensure that each joint was fully mobilized. The purpose of massage and PROM is to prevent muscle contractures and joint stiffness.

Starting from 72 h after surgery, with the help of a passive standing device, the dog was allowed to make a correct standing posture, subject to the dog’s ability to tolerate, usually for 1–3 min each time, 3–4 times a day, and the time was increased with the gradual recovery of the dog, in order to accelerate the repair of neural plasticity and to improve the dog’s ability to perceive the position of the limbs.

**Figure 2:**
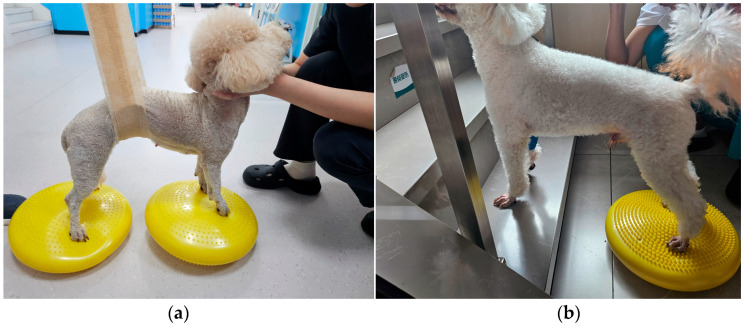
(**a**) Assisted standing on a balance board; (**b**) Hind limb strengthening training.

Subsequently, a rough-surfaced inflatable balance board was used to have the dog stand on the board. Initially, assistance may be provided (Figure 2a). Each stay lasted 5–10 s, and as the dog gradually recovered, the duration is gradually increased. Light pressure is applied to one side of the balance board to induce the dog to actively adjust its posture to maintain balance. For dogs with significant hind limb weakness, training is focused on the hind limbs (Figure 2b), with the front limbs placed on a step and the hind limbs on the balance board, while applying light pressure to the balance board. Training was alternated between both sides, with 3–5 sessions per day. This promotes reflex integration and neuromuscular adaptive conditioning.

**Figure 3:**
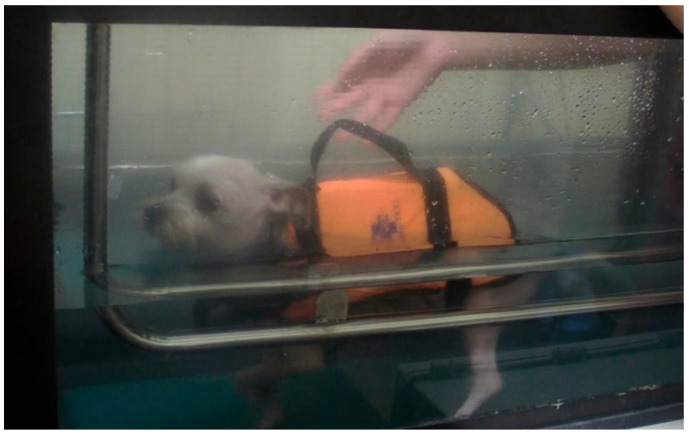
UWTM training of the affected dog.

When the dog’s skin incision healed and it was able to walk to some extent, UWTM training was performed (Figure 3). The water temperature was controlled at about 26 °C. The duration of training was gradually increased from 5 min per session to 1 h per day, and the speed was gradually increased from 0.5 km/h (0.14 m/s) to 3 km/h(0.83 m/s), 1–2 days per week, and it was necessary to avoid contamination of the surgical incision with water during the training process.

The training environment was always quiet, supplemented by soft music, and sufficient stimulation was provided to stimulate the willingness to exercise when necessary. Vital signs were closely monitored during the training.

### 2.5. Statistical Analysis

Continuous variables were assessed for normality using the Shapiro–Wilk test. Data with normal distribution are presented as mean ± standard deviation (Mean ± SD), while non-normally distributed data are expressed as median and interquartile range (M (Q1, Q3)). Categorical variables are summarized as counts and percentages (*n*, %). All hypothesis tests were two-tailed, and a *p* < 0.05 was considered statistically significant.

To control for potential confounding bias between groups, a stratified propensity score matching (PSM) analysis was employed. The specific strategy involved first stratifying the cohort based on Rusbridge grade (Grades 2, 3, and 4). Within each stratum, PSM was performed independently to ensure comparability of baseline characteristics between the ALRG and LRG groups across all subgroups. The propensity score was calculated using a binary logistic regression model that included covariates such as breed, age, sex, body weight, and location of the herniated disc. The matching process utilized a nearest-neighbor 1:1 matching algorithm with a caliper width of 0.02 to exclude poorly matched pairs and ensure matching quality.

Before matching, between-group comparisons of continuous variables were performed using the independent samples t-test for normally distributed data with homogeneous variance and the Mann–Whitney U test for non-normally distributed data. Categorical variables were compared using Pearson’s chi-square test or Fisher’s exact test, as appropriate. After matching, continuous variables were compared using the paired samples t-test (normal distribution) or the Wilcoxon signed-rank test (non-normal distribution). Categorical variables were compared using McNemar’s test for binary outcomes and the McNemar-Bowker test for multinomial outcomes. For primary outcomes (time to standing and walking recovery) and continuous secondary outcomes (e.g., Olby scores at various time points), the mean difference (MD) with its 95% confidence interval (95% CI) was reported for normally distributed data, while the median difference with its 95% CI was reported for non-normally distributed data. For binary outcomes, the risk difference (RD) with its 95% CI was reported.

## 3. Results

### 3.1. Baseline Characteristics

During the study period, a total of 136 dogs with cervical IVDH underwent surgical treatment and postoperative rehabilitation, of which 79 met the inclusion criteria. After propensity score matching stratified by Rusbridge grade, 40 dogs were successfully matched. The matching results for each grade are presented in Table 2, and the detailed breed distribution is provided in Table A2.

In the Rusbridge grade 2 subgroup, formal statistical comparisons were not performed due to the limited sample size. After matching, each group contained 3 dogs. Descriptive data indicated that the ALRG group had higher mean age (7.33 ± 2.52 years vs. 7.00 ± 2.65 years) and body weight (4.62 ± 2.39 kg vs. 3.27 ± 1.76 kg) compared to the LRG group, while sex distribution was identical (3 males in each group, 100%). Regarding lesion location, both groups showed identical involvement at C3–C4 (33.33% each). The ALRG group exhibited lesions at C4–C5 (33.33%) and C6–C7 (33.33%), whereas the LRG group showed a higher proportion of involvement at C6–C7 (66.67%). Both groups were predominantly Poodles before and after matching.

Among Rusbridge grade 3 cases, the ALRG group (*n* = 17) and LRG group (*n* = 10) showed no statistically significant differences in baseline characteristics before matching. After matching, 10 dogs were included in each group, with well-balanced covariates: the ALRG group was slightly older (7.60 ± 3.20 years vs. 7.10 ± 3.11 years, *p* = 0.747), while the LRG group had greater body weight (6.91 ± 3.45 kg vs. 5.48 ± 2.31 kg, *p* = 0.190). The LRG group had a higher proportion of males (50% vs. 30%, *p* = 0.625). The distribution of lesion sites was similar (*p* = 0.156), with a slightly higher proportion of C3–C4 and C4–C5 lesions in the ALRG group. Both groups were primarily composed of Poodles (ALRG: 50% vs. LRG: 30%), and the ALRG group had a higher percentage of Bichon Frisés (30% vs. 0%).

For Rusbridge grade 4 dogs, a significant difference in age was observed before matching between the ALRG (*n* = 20) and LRG (*n* = 13) groups, with the ALRG group being significantly older (7.00 [4.50–9.00] years vs. 4.00 [3.00–6.50] years, *p* = 0.047). After matching, each group contained 7 dogs, and this difference was eliminated (*p* = 0.253). The LRG group had a higher mean body weight (12.35 ± 7.45 kg vs. 8.46 ± 5.24 kg, *p* = 0.393), while sex distribution was identical. The ALRG group had a higher proportion of lesions at C2–C3 (57.14% vs. 42.86%), whereas the LRG group showed a higher proportion at C4–C5 (14.29% vs. 0%). Mixed-breed dogs were most common in both groups.

### 3.2. Primary Efficacy Endpoint

**Figure 4:**
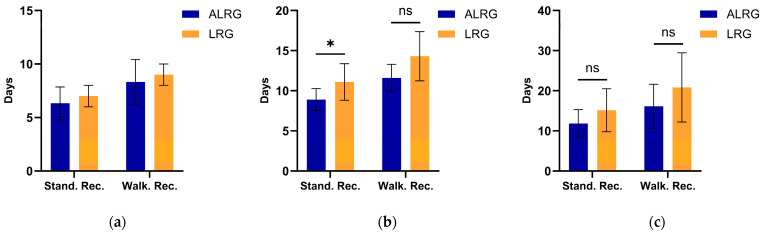
Comparison of standing and walking recovery times between groups after matching. (**a**) Rusbridge Grade 2, (**b**) Rusbridge Grade 3, and (**c**) Rusbridge Grade 4 dogs. Data are presented as mean ± standard deviation. NS, *p* > 0.05; *, *p* < 0.05. Formal statistical testing was not performed for grade II subgroups (*n* = 3 per group) due to small sample size. Stand. Rec., standing recovery time; Walk. Rec., walking recovery time.

Analysis after PSM revealed significantly shorter standing and walking recovery times in the ALRG group, a trend that remained consistent across all Rusbridge grades (Table 3, Figure 4). In Grade III dogs, the ALRG group demonstrated a shorter mean standing recovery time compared to the LRG group (8.90 ± 1.37 days vs. 11.10 ± 2.28 days; MD, −2.20 days; 95% CI, −4.38 to −0.02; *p* = 0.048). A similar advantage was observed in walking recovery, with the ALRG group showing a notable reduction in time (11.60 ± 1.71 days vs. 14.30 ± 3.05 days; MD, −2.70 days; 95% CI, −5.42 to 0.02; *p* = 0.051). Among Grade IV dogs, the ALRG group continued to exhibit shorter mean times for both standing (11.86 ± 3.44 days vs. 15.14 ± 5.37 days; MD, −3.29 days) and walking recovery (16.14 ± 5.46 days vs. 20.56 ± 8.63 days; Median Difference, −4 days), although these differences did not reach statistical significance (*p* = 0.250 and *p* = 0.396, respectively). For Grade II dogs, descriptive data also indicated a pattern of shorter recovery times in the ALRG group for both standing (6.33 ± 1.53 days vs. 7.00 ± 1.00 days) and walking (8.33 ± 2.08 days vs. 9.00 ± 1.00 days); however, formal comparison was precluded by the small matched cohort size (*n* = 3 per group).

**Figure 5:**
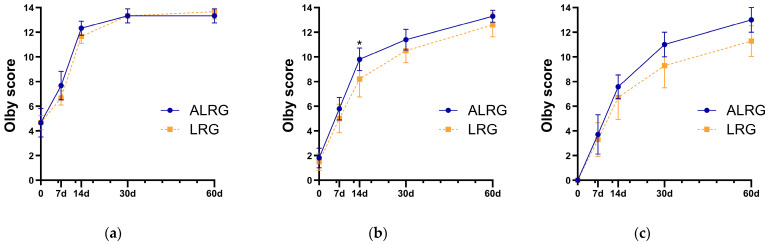
Comparison of postoperative Olby scores at various time points between groups after matching. (**a**) Rusbridge Grade 2, (**b**) Rusbridge Grade 3, and (**c**) Rusbridge Grade 4 dogs. Data are presented as mean ± standard deviation. *, *p* < 0.05.

In addition, after PSM, Olby scores of dogs in the ALRG group with different Rusbridge gradings were compared with those of dogs in the LRG group at pre- and postoperative time points (7, 14, 30, and 60 days) (Table 4, Figure 5). In Rusbridge grade 2 cases, statistical comparisons were not performed due to limited sample size, though the ALRG group showed marginally lower scores at 60 days (13 vs. 14). For Rusbridge grade 3 dogs, preoperative scores were comparable between groups (MD: 0.30, *p* =0.434). The ALRG group demonstrated significantly better recovery at 14 days (MD: 1.60, *p* = 0.029). Although not statistically significant, the ALRG group maintained higher scores at other timepoints—1 point higher at 7 days, 0.90 at 30 days, and 0.70 at 60 days. Rusbridge Grade 4 cases showed the most consistent trend: the ALRG group outperformed controls by 0.43 points at 7 days, 0.86 at 14 days, and notably by 1.71 points at both 30 days (*p* = 0.053) and 60 days (*p* = 0.061), with the latter two timepoints approaching significance.

### 3.3. Secondary Efficacy Endpoint

We compared analgesic utilization patterns between the ALRG and LRG groups across Rusbridge grades after PSM (Table 5). With the exception of the Rusbridge grade 2 subgroup, for which formal statistical comparison was not performed due to small sample size, no statistically significant differences were observed in the utilization rates of the three classes of analgesic drugs between the two groups at other grades (*p* > 0.05). Specifically, the utilization rates and RD for calcium channel modulators were highly consistent across all grades and overall. NSAIDs demonstrated identical utilization rates (100%) in most tiers. Regarding treatment duration, the two groups also exhibited consistent patterns in most instances. For NSAIDs and calcium channel modulators, there were no statistically significant differences (*p* > 0.05) in treatment duration across various Rusbridge grades or overall. However, it is noteworthy that the opioid treatment duration was significantly shorter in the ALRG group compared to the LRG group at Rusbridge grade 3 (MD: −2.20, 95% CI: −3.40 to −0.99, *p* = 0.003) and overall (MD: 0.20, 95% CI: 0.10 to 0.31, *p* = 0.001). Furthermore, a trend towards shorter opioid treatment duration in the ALRG group was observed in patients with Rusbridge grade 4 (MD: −1.71, 95% CI: −3.83 to 0.40, *p* = 0.095). A trend towards reduced use of NSAIDs was also generally present in the ALRG group, although the differences did not reach statistical significance.

Long-term prognosis follow-up achieved a 100% response rate. Comparative results in Long-term prognosis between the ALRG and LRG groups after PSM are summarized for the overall cohort and by Rusbridge grade in Table 6. In the overall cohort, a statistically significant difference was observed in the distribution of long-term prognosis outcomes between the two groups (*p* = 0.048). Specifically, the proportion of patients achieving complete success was significantly higher in the ALRG group compared to the LRG group (80% vs. 50%). Furthermore, no patients in the ALRG group were categorized as minimal improvement, whereas 25% of patients in the LRG group fell into this category. In the Rusbridge grade 3 and 4 subgroups, descriptive comparisons showed a trend toward better outcomes in the ALRG group. Among grade 3 cases, 90% of ALRG patients achieved complete success compared to 50% in the LRG group. In grade 4 cases, 57.14% of ALRG patients achieved complete success versus 28.57% in the LRG group. However, formal statistical comparisons were not performed for these subgroups due to complete separation in outcome categories. In the Rusbridge grade 2 subgroup (ALRG *n* =3, LRG *n* = 3), all patients in both groups achieved complete success (100% vs. 100%), with no cases of partial success or minimal improvement observed. Statistical comparison was not performed for this subgroup due to the small sample size.

## 4. Discussion

This study compared the rehabilitation outcomes between dogs treated with AM combined with LT versus dogs receiving LT alone following cervical IVDH decompression surgery. A total of 79 dogs were enrolled. The inclusion of only cervical segments was due to differences in surgical techniques and acupoint selection for AM compared to thoracolumbar IVDH. Dogs were assigned to either the ALRG group or the LRG group based on their owners’ choice of rehabilitation protocol postoperatively. Both groups received identical diagnostic and treatment protocols prior to rehabilitation therapy, including MRI imaging, VSD surgery, and nutritional care. All dogs initiated rehabilitation therapy within a 24- to 72-h postoperative window after achieving stable vital signs and effective acute pain control. No significant differences were observed between groups in gender, age, or weight distribution (Table 2), ensuring comparability between the two cohorts.

Dogs were assigned to either the ALRG group or the LRG group based on owner choice rather than random allocation. We acknowledge that this design theoretically introduces selection bias, as owners choosing the ALRG group may differ from those selecting the traditional rehabilitation group due to personal beliefs, economic circumstances, or preferences for integrative therapy. To minimize this bias and ensure comparability between groups at baseline, we employed PSM. All recorded covariates potentially affecting prognostic outcomes were included, including age, weight, sex, affected disc level, and breed. Matching was performed based on these covariates, and the final sample analyzed for outcome measures comprised the matched cohort. Crucially, after matching, no significant differences were observed across variables in any subgroup except the Grade 2 subgroup, where statistical testing was not performed due to insufficient sample size. Although the breed variable in the Grade 3 and 4 subgroups and the lesion location variable in the Grade 3 subgroup had insufficient sample sizes and numerous classification categories, resulting in excessively low expected cell frequencies in the contingency table for effective statistical testing, their descriptive distributions showed high consistency between the two groups. This outcome indicates that we successfully achieved a dataset with high comparability across known potential confounders, thereby effectively simulating a randomized controlled trial environment. Of course, as with any observational study, the influence of unmeasured potential confounders cannot be entirely ruled out.

Based on data from 79 affected dogs prior to matching (Table 2, Table A2), the pooled analysis revealed that males constituted 64.56% (*n* = 51) and females 35.44% (*n* = 28). While castration status was not recorded in this study, the significantly higher incidence among males aligns with previous research. The mean age of all dogs was 6.89 ± 3.14 years, with a mean weight of 7.75 ± 7.52 kg. Regarding affected segments, C2-C3 (*n* = 26) was the most common IVDH location in this study, followed by C3-C4 (*n* = 20). This distribution also aligns with most literature reports [9,14,19]. However, some reports indicate C6-C7 as the most frequent site [1,12]. Regarding breed distribution, Poodles were the most prevalent breed, accounting for 26.58% (*n* = 21), followed by mixed-breed dogs at 11.39% (*n* = 9) and Bichon Frisés at 7.59% (*n* = 6). This differs from previous studies frequently reporting breeds such as Dachshunds [19], Shih Tzus [23], Doberman Pinschers [43], Maltese [44], French Bulldogs [44], and mixed-breed dogs [45]. Notably, we identified the first documented cases of Chinese Rural Dog (*n* = 6). This finding is likely due to breed distribution patterns in Northeast China rather than breed susceptibility.

The modified Rusbridge grading system used in this study is primarily based on motor functional status. Motor dysfunction may subsequently lead to factors such as muscle atrophy, joint stiffness, and neurogenic bladder, and some clinical studies have shown that preoperative motor functional status is significantly correlated with postoperative rehabilitation outcomes [46,47]. In particular, ventilatory dysfunction may be correlated with the severity of neurological injury [19]. The modified Rusbridge grading system is effective in predicting the rehabilitation outcome after canine IVDH, and its assessment efficacy has been validated in several clinical studies [9,20,22,24]. The results of this study further corroborate the effectiveness of the Rusbridge grading system as a prognostic tool for canine IVDH. An important finding was that the recovery of motor function, dependence on analgesic medication, and long-term therapeutic outcomes all exhibited a gradient significantly associated with the initial Rusbridge grade.

The fundamental purpose of applying the Olby scoring system—originally designed and validated for thoracolumbar spinal cord injuries—to evaluate postoperative rehabilitation in cervical IVDH is to objectively quantify the sequential stages of motor function recovery from paralysis to normal gait. This approach may be conceptually applicable to spinal cord injuries at different anatomical levels. It must be noted that this scale does not account for certain cervical-specific deficits caused by cervical spinal cord injury, such as neck pain or isolated forelimb dysfunction. Therefore, in this study, the Olby score serves solely as a continuous indicator measuring the overall recovery of motor function and gait ability. The dynamic changes observed in this metric correlate closely with reduced recovery times for standing and walking, thereby facilitating a comprehensive assessment of the rehabilitation status of motor function.

Although existing research has confirmed the clear clinical value of rehabilitation therapy for dogs following surgery for intervertebral disc disease, its efficacy remains significantly limited in severe cases. Gouveia et al. [22] found that among quadriplegic dogs following cervical disc disease surgery, those exhibiting spinal cord hyperexcitability demonstrated significantly poorer recovery of walking ability, increased incidence of new excitatory symptoms, and fewer days of ambulatory function compared to non-excitable dogs. This suggests rehabilitation protocols may be insufficient for treating severe cases. Jeong et al. [23] further revealed a negative correlation between rehabilitation efficacy and disease severity: Although the rehabilitation group achieved high success rates for Rusbridge grades 1–3 (100%, 100%, and 75%), the success rate for grade 4 cases dropped to 53.85%. While this was significantly better than the non-rehabilitation group (28.57%), half of the animals still failed to recover. This limitation highlights the current rehabilitation protocols’ effectiveness constraints for cases with severe neurological deficits.

AM therapies usually target the underlying etiology of the disease to achieve efficacy in eliminating its root cause. The AM acupoint selection protocol in this study integrated TCVM with modern neuroscience, drawing on meridian-based acupoints from TCVM and supplementing them with evidence-based acupoints. Within the meridian-based framework, Jing-Jiaji was selected as a direct pathway to treat the disease, along with BL-11, BL-20, BL-23, and BL-18 on the bladder meridian, which together formed a “dorsal yu acupoint pairing” to achieve both symptomatic and radical treatment. In accordance with the traditional Chinese medical principle of “treating impotence by taking Yangming alone,” acupoint ST-36 was also included. Additional acupoints used in this study included LU-7, which is indicated for nuchal rigidity; LI-4 and LIV-3, known as the “Four Guan Points” for soothing the liver and relieving depression; KID-3, an original point of the Kidney Meridian reflecting the theory that “the Kidney governs bones and marrow” to tonify innate constitution; and GB-20, a point from the Shao-Yang meridian used to pacify the liver and calm the spirit.

In this study, EA employed high-frequency EA stimulation at 40–80 Hz, as it has been shown to effectively promote local microcirculation [48], facilitating the clearance of inflammatory substances while simultaneously inhibiting pain signal transmission [49]. It also significantly promotes peripheral nerve repair to a certain extent [50]. Compared to low-frequency EA, high-frequency stimulation more readily induces rhythmic muscle contractions, which may better improve disuse muscle atrophy and enhance proprioceptive recovery. Considering anatomical locations, the GB-20 and LIV-3 acupoints were deemed unsuitable for EA. GB-20 is situated between the atlas and axis vertebrae, adjacent to the medulla oblongata, where electrical diffusion could potentially interfere with brainstem function. LIV-3 lies within the sensitive interdigital region, where high nerve density reduces tolerance to electrical stimulation and may induce agitation in animals. Additionally, research by Pei et al. [51] indicates that MA stimulation of GB-20 increases vertebral artery blood flow velocity. This may help alleviate neck muscle tension and spasticity, facilitate the removal of inflammatory metabolites, reduce nerve root edema, and deliver nutrients to damaged tissues.

Clinical practice regarding canine IVDH has shown that early LT at 24 h postoperatively is not only safe, but also promotes functional recovery [22,52,53,54]. In the pre-sent study, PROM was used with the aim of improving joint range of motion and muscle function, assisted standing training in order to enhance proprioceptive input and neuromuscular activation, and balance board training to improve postural reflexes. According to the current clinical consensus, the early intervention of PROM, assisted standing training, and balance board training at 24 h postoperatively is effective [55].

LT promotes the reorganization of neural networks after the loss of locomotor ability and neuronal damage [56,57]. UWTM is a tool for providing this type of exercise to dogs and is now one of the core protocols for postoperative rehabilitation of canine IVDH [23,54,58]. Most protocols support UWTM training 5 days per week, but in the present study, considering that UWTM may affect the internal regulation of the body after AM and in order to prevent the transition of the canine’s energy expenditure, the training of UWTM 1–2 days per week was used and was performed 48 h apart from the AM treatment.

In this study, comparing the time to stand recovery and time to walk recovery between the two groups of dogs (Table 3, Figure 4), we observe that the ALRG group consistently demonstrated superior motor function recovery compared to the LRG group, with the difference becoming more pronounced in dogs with higher Rusbridge grades. Although statistical comparisons were not performed for Grade 2 cases due to sample size limitations, their mean values already indicated a difference favoring the ALRG group. In Grade 3 cases, the reduction in standing recovery time reached statistical significance (MD: −2.20 days, *p* = 0.048), while the difference in walking recovery time approached significance (MD: −2.7 days, *p* = 0.051). Most importantly, in Grade 4 cases, the ALRG group demonstrated a more pronounced advantage in both standing and walking recovery times (MD: −3.29 days and Median Difference: −4 days). Although the difference did not reach statistical significance, this may be attributable to the small sample size (ALRG Group: *n* = 7; LRG Group: *n* = 7).

The dynamic changes in Olby scores (Table 4, Figure 5) further validated this pattern: by the early postoperative period (Day 7), the ALRG group in Grade 3 cases already showed a trend toward higher scores (Median Difference: 1). By Day 14 postoperatively, this scoring advantage in Grade 3 cases had evolved into a statistically significant difference (MD: 1.60, *p* = 0.029). For Grade 4 cases, this trend of widening disparity over time was even more pronounced. Although differences were not yet significant at days 7 and 14, the ALRG group demonstrated a substantial scoring advantage starting from day 30 (MD: 1.71, *p* = 0.053), which had persisted through day 60 (MD: 1.71, *p* = 0.061).

We believe that the superior motor function recovery observed in the ALRG group compared to the LRG group may primarily be attributed to the efficacy of AM. Research indicates that EA can restore control over intact neuromuscular systems by enhancing spinal cord signal processing capabilities and regulating neural activity in the motor and sensory cortices [33]. This contributes to improved neuromuscular function in dogs with spinal cord injuries. Moxibustion exerts therapeutic effects through multiple mechanisms, including promoting blood circulation and alleviating inflammatory responses. Anatomical findings reveal a rich distribution of nerves and blood vessels around the ST-36 acupoint. Moxibustion applied to this point after acupuncture may further aid in warming yang and dispelling cold, thereby improving blood circulation. This effectively alleviates symptoms such as pain, stiffness, and weakness in the lower limbs. Additionally, the LI-4, LU-7, ST-36, LIV-3, and KID-3 acupoints are all located at the extremities of the limbs. These points exhibit high overlap with the distribution of superficial cutaneous nerves and deep muscle spindle and tendon receptors. Stimulating peripheral nerve fibers can influence the upward transmission of proprioceptive signals [59], thereby facilitating the recovery of proprioception in dogs following cervical IVDH surgery.

Postoperative pain management is a critical component of neurosurgical rehabilitation, aiming to achieve effective analgesia while minimizing medication-related side effects. Analysis of analgesic usage in this study revealed potential advantages of combining AM with LT therapy. The most prominent finding was a significant and consistent reduction in opioid duration in the ALRG group (Table 5). Across all Rusbridge Grades, the median opioid duration in the ALRG group was 1.6 days shorter than in the LRG group, with this difference being highly significant (*p* = 0.001). This suggests that AM intervention may reduce opioid requirements in dogs through its analgesic effects, which is significant for mitigating common side effects such as respiratory depression associated with these drugs. Acupuncture has been demonstrated to enhance analgesia in dogs with intervertebral disc disease [60]. It also shows efficacy in postoperative pain management; for instance, Luna et al. found it comparable to morphine or carprofen for analgesia following ovariohysterectomy in dogs [61].

Additionally, data in Table 5 suggest a potential positive trend in NSAID usage within the ALRG group. Although no statistically significant differences were observed in overall usage rates or duration across all severity levels, the mean duration of NSAID use was shorter in the ALRG group compared to the LRG group for both Grade 2 and Grade 3 cases (e.g., Grade 3 cases MD: −0.60 days). Although this effect size was small and non-significant, its consistent directionality suggests that AM may offer benefits in alleviating inflammatory pain, potentially reducing reliance on NSAIDs to some extent. Regarding calcium channel modulators, usage patterns were highly similar across both groups. This may reflect either minimal influence of AM on usage patterns for this class of drugs or inferior neuro-regenerative effects of the high-frequency EA used in our study compared to low-frequency EA.

Long-term prognosis (Table 6) revealed that among all dogs with Rusbridge Grade disease, the proportion of complete success cases in the ALRG group (80%) was significantly higher than that in the LRG group (50%) (*p* = 0.048). Furthermore, the disparity increased with higher Rusbridge Grade. The most pronounced difference was observed in Grade 4 dogs (57.14% vs. 28.57%). Notably, no dogs in the ALRG group experienced the adverse outcome of “minimal improvement,” whereas 5 dogs (25%) in the LRG group with grades 3–4 exhibited minimal improvement. As shown in Table 3, all dogs regained the ability to walk more than 10 steps without assistance in the short term. This finding is inconsistent with the Long-term prognosis results in the LRG group, where minimal improvement emerged at 6–8 months. Argent et al. [14] reported a recurrence rate as high as 33% following canine cervical IVDH surgery, suggesting recurrence may explain this outcome. This indicates that early recovery of motor function does not equate to stable Long-term prognosis. Although our follow-up achieved a 100% response rate, minimizing nonresponse bias to the greatest extent possible, it must be acknowledged that we did not employ validated standardized functional scales. Consequently, potential recall bias could not be avoided. Future rehabilitation studies should prioritize evaluating long-term outcomes using validated standardized functional scales. An ideal postoperative rehabilitation protocol for canine cervical IVDH should minimize this phenomenon.

Rodent studies confirm that the BL-23 acupoint shares common sensory and sympathetic innervation with the kidneys, and stimulation of BL-23 can influence bladder smooth muscle activity [62]. Human clinical studies further demonstrate that stimulating BL-23 improves urinary frequency and urinary retention [63,64]. Stimulation of KID-3 reduces bladder C-fiber activity, thereby inhibiting urinary incontinence [65]. We consider that these two acupoints may be beneficial for preventing postoperative neurogenic bladder or promoting its recovery. Unfortunately, this retrospective study did not record relevant indicators.

The synergistic effect of AM combined with LT may also be a key reason for the superior efficacy of the ALRG group over the LRG group. The analgesic and anti-inflammatory effects of acupuncture enable dogs to commence exercise training earlier and with greater efficiency. A meta-analysis of human spinal cord injury [66] demonstrated that EA combined with exercise significantly outperformed exercise alone in restoring motor function. This combined therapy is now widely applied in human medicine for conditions such as stroke-induced hemiplegia [67,68], knee osteoarthritis [69,70], and cervical-shoulder syndrome [71].

Based on the findings of this study, we believe that for severe cases of IVDH, rehabilitation therapy combining AM with LT following VSD is particularly suitable. Previous studies on long-term conservative AM treatment for canine IVDH have also demonstrated certain improvement effects, enabling some affected dogs to recover to a stable condition. [29,30,60]. However, we consider that AM cannot directly remove the protruding disc material, though it can improve microcirculation and reduce edema. Prolonged AM conservative treatment may exacerbate muscle atrophy. Furthermore, cervical IVDH often causes acute mechanical compression of the spinal cord, whereas VSD can immediately relieve compression, preventing irreversible neurological damage.

A retrospective study by Joaquim et al. [32] compared the efficacy of decompression surgery alone, conservative EA treatment alone, and EA following decompression surgery for canine IVDH. The results revealed that dogs receiving EA alone achieved a higher rate of clinical success than those undergoing decompression surgery followed by EA. It should be noted that this study employed low-frequency EA (5/15 Hz) with specific point combinations, primarily targeting rehabilitation in chronic cases. In contrast, the present study utilized high-frequency EA (40–80 Hz) with specific point combinations, primarily aimed at postoperative multifunctional reconstruction. However, the present study did not establish a group receiving conservative AM therapy alone, thus preventing direct comparison of the efficacy differences between surgery combined with AM and AM alone.

This study has several limitations. First, regarding the research design, although PSM was employed to enhance comparability between groups and control for known confounding factors, as a retrospective study, data collection remains constrained by the completeness of medical records. Additionally, potential factors such as socioeconomic background and cognitive preferences may influence owners’ decisions regarding nutritional supplements and other interventions for their animals, and these confounding variables could still interfere with the results to some extent. Furthermore, multiple veterinarians participated in the dogs’ treatment and care, potentially affecting the reliability of the findings. Second, regarding efficacy assessment, this study primarily focused on indicators related to motor function recovery, lacking documentation of prognostic indicators such as autonomic function, respiratory function, and sensory abnormalities. Additionally, the study sample size was relatively small, particularly with a low proportion of Rusbridge Grade 2 cases, which may have limited the analysis of efficacy differences. Future studies are recommended to adopt a prospective design to comprehensively evaluate the rehabilitation efficacy of AM combined with LT following cervical IVDH surgery in dogs.

## 5. Conclusions

This study represents a preliminary exploration, marking the first application of combined AM and LT for postoperative rehabilitation in dogs with cervical IVDH. The results indicate that this combined approach demonstrates good clinical feasibility. Dogs receiving AM therapy exhibited shorter recovery times for standing and walking postoperatively, showed a more favorable trend in Olby score improvement across five postoperative time points, required reduced postoperative opioid usage, and achieved better long-term neurological outcomes. These findings provide valuable methodological insights and preliminary data support for developing novel rehabilitation protocols. However, prospective studies are warranted for further investigation.

## Figures and Tables

**Table 1:** Rusbridge’s modified canine neurologic dysfunction grading system.

Rusbridge Grade	
Grade	Description
0	Normal neurologic status
1	Ambulatory with mild proprioceptive and motor deficits
2	Ambulatory with moderate proprioceptive and motor deficits
3	Ambulatory with severe proprioceptive and motor deficits
4	Nonambulatory tetraparesis or tetraplegia without respiratory compromise
5	Tetraplegia with respiratory impairment

**Table 2:** Baseline Characteristics Before and After PSM Conducted Within Each Rusbridge Grade.

	Before Matching			After Matching		
**Rusbridge Grade 2**						
	ALRG (*n* = 10)	LRG (n = 9)	*p*	ALRG (*n* = 3)	LRG (*n* = 3)	*p*
Age, years	8.10 ± 3.70	6.56 ± 3.13	‡	7.33 ± 2.52	7.00 ± 2.65	‡
Weight, kg	3.45 (1.97–7.46)	2.42 (2.18–9.55)	‡	4.62 ± 2.39	3.27 ± 1.76	‡
Sex			‡			‡
Male	6 (60%)	7 (77.78%)		3 (100%)	3 (100%)	
Female	4 (40%)	2 (22.22%)		0 (0%)	0 (0%)	
IVDH Localization			‡			‡
C2-C3	3 (30%)	0 (0%)		0 (0%)	0 (0%)	
C3-C4	3 (30%)	2 (22.22%)		1 (33.33%)	1 (33.33%)	
C4-C5	1 (10%)	2 (22.22%)		1 (33.33%)	0 (0%)	
C5-C6	0 (0%)	0 (0%)		0 (0%)	0 (0%)	
C6-C7	3 (30%)	5 (55.56%)		1 (33.33%)	2 (66.67%)	
Breed			‡			‡
(See Appendix A Table A2)	Mixed	Mixed		Mixed	Mixed	
**Rusbridge Grade 3**						
	ALRG (*n* = 17)	LRG (*n* = 10)	*p*	ALRG (*n* = 10)	LRG (*n* = 10)	*p*
Age, years	7.06 ± 2.84	7.10 ± 3.11	0.972	7.60 ± 3.20	7.10 ± 3.11	0.747
Weight, kg	5.14 (4.17–8.89)	5.86 (4.38–8.61)	0.651	5.48 ± 2.31	6.91 ± 3.45	0.190
Sex			1.000			0.625
Male	9 (52.94%)	5 (50%)		3 (30%)	5 (50%)	
Female	8 (47.06%)	5 (50%)		7 (70%)	5 (50%)	
IVDH Localization			1.000			0.156
C2-C3	3 (17.65%)	2 (20%)		3 (30%)	2 (20%)	
C3-C4	4 (23.53%)	2 (20%)		2 (20%)	2 (20%)	
C4-C5	4 (23.53%)	3 (30%)		2 (20%)	3 (30%)	
C5-C6	3 (17.65%)	2 (20%)		1 (10%)	2 (20%)	
C6-C7	3 (17.65%)	1 (10%)		2 (20%)	1 (10%)	
Breed			0.173			N/A
(See Appendix A Table A2)	Mixed	Mixed				
**Rusbridge Grade 4**						
	ALRG (*n* = 20)	LRG (*n* = 13)	p	ALRG (*n* = 7)	LRG (*n* = 7)	*p*
Age, years	7.00 (4.50–9.00)	4.00 (3.00–6.50)	0.047	7.71 ± 3.64	6.43 ± 3.55	0.253
Weight, kg	5.95 (3.68–11.70)	10.10 (5.03–12.59)	0.217	8.46 ± 5.24	12.35 ± 7.45	0.393
Sex			1.000			1.000
Male	14 (70%)	10 (76.92%)		5 (71.43%)	5 (71.43%)	
Female	6 (30%)	3 (23.08%)		2 (28.57%)	2 (28.57%)	
IVDH Localization			0.488			N/A
C2-C3	13 (65%)	5 (38.46%)		4 (57.14%)	3 (42.86%)	
C3-C4	3 (15%)	5 (38.46%)		2 (28.57%)	2 (28.57%)	
C4-C5	1 (5%)	1 (7.69%)		0 (0%)	1 (14.29%)	
C5-C6	2 (10%)	1 (7.69%)		1 (14.29%)	1 (14.29%)	
C6-C7	1 (5%)	1 (7.69%)		0 (0%)	0 (0%)	
Breed			0.550			N/A
(See Appendix A Table A2)	Mixed	Mixed		Mixed	Mixed	

Legend: Data are presented as mean ± standard deviation, median (interquartile range), or n (%). ‡ *p* value not reported for the Rusbridge Grade 2 subgroup due to small sample size, which precluded meaningful statistical testing. N/A Not applicable; a valid statistical test could not be performed due to complete separation or lack of variability in the data after matching.

**Table 3:** Comparison of Standing and Walking Recovery Times Between ALRG and LRG Groups After PSM, Stratified by Rusbridge Grade.

Variable	Rusbridge Grade	Group	Days	Effect Size (95% CI)	*p*
Standing recovery time	2	ALRG (*n* = 3)	6.33 ± 1.53	‡	‡
		LRG (*n* = 3)	7.00 ± 1.00		
	3	ALRG (*n* = 10)	8.90 ± 1.37	MD: −2.20 (−4.38, −0.02)	0.048
		LRG (*n* = 10)	11.10 ± 2.28		
	4	ALRG (*n* = 7)	11.86 ± 3.44	MD: −3.29 (−9.60, 3.03)	0.250
		LRG (*n* = 7)	15.14 ± 5.37		
Walking recovery time	2	ALRG (*n* = 3)	8.33 ± 2.08	‡	‡
		LRG (*n* = 3)	9.00 ± 1.00		
	3	ALRG (*n* = 10)	11.60 ± 1.71	MD: −2.70 (−5.42, 0.02)	0.051
		LRG (*n* = 10)	14.30 ± 3.05		
	4	ALRG (*n* = 7)	16.14 ± 5.46	Median Difference: −4 (−12, 4)	0.396
		LRG (*n* = 7)	20.56 ± 8.63		

Legend: Data are presented as mean ± standard deviation. Effect sizes are reported as Mean Difference (MD) with 95% confidence interval derived from paired t-test for parametric data, or as Median Difference with 95% bootstrap confidence interval for non-parametric data. ‡ *p* value not reported for the Rusbridge Grade 2 subgroup due to small sample size, which precluded meaningful statistical testing.

**Table 4:** Comparison of Dynamic Changes in Olby Scores from Preoperative to 60 Days Postoperative Between ALRG and LRG Groups After PSM, Stratified by Rusbridge Grade.

Rusbridge Grade	Time Point	Group	Olby Score	Effect Size (95% CI)	*p*
2	0 d	ALRG (*n* = 3)	4 (4–5)	‡	‡
		LRG (*n* = 3)	5 (4–5)		
	7 d	ALRG (*n* = 3)	7 (7–8)	‡	‡
		LRG (*n* = 3)	7 (6.5–7)		
	14 d	ALRG (*n* = 3)	12 (12–12.5)	‡	‡
		LRG (*n* = 3)	12 (11.5–12)		
	30 d	ALRG (*n* = 3)	13 (13–13.5)	‡	‡
		LRG (*n* = 3)	13 (13–13.5)		
	60 d	ALRG (*n* = 3)	13 (13–13.5)	‡	‡
		LRG (*n* = 3)	14 (13.5–14)		
3	0 d	ALRG (*n* = 10)	1.80 ± 0.79	MD: 0.30 (−0.53, 1.13)	0.434
		LRG (*n* = 10)	1.50 ± 0.71		
	7 d	ALRG (*n* = 10)	5.80 ± 0.29	Median Difference: 1 (0–2)	0.131
		LRG (*n* = 10)	5.00 ± 0.37		
	14 d	ALRG (*n* = 10)	9.80 ± 0.92	MD: 1.60 (0.20,3.00)	0.029
		LRG (*n* = 10)	8.20 ± 1.48		
	30 d	ALRG (*n* = 10)	11.40 ± 0.84	MD: 0.90 (−0.29, 2.09)	0.121
		LRG (*n* = 10)	10.50 ± 0.97		
	60 d	ALRG (*n* = 10)	13.30 ± 0.48	MD: 0.70 (−0.20, 1.60)	0.111
		LRG (*n* = 10)	12.60 ± 0.97		
4	0 d	ALRG (*n* = 7)	0.00 ± 0.00	MD: 0	N/A
		LRG (*n* = 7)	0.00 ± 0.00		
	7 d	ALRG (*n* = 7)	3.71 ± 1.60	MD: 0.43 (−1.25, 2.11)	0.555
		LRG (*n* = 7)	3.29 ± 1.38		
	14 d	ALRG (*n* = 7)	7.57 ± 0.98	MD: 0.86 (−1.03, 2.74)	0.308
		LRG (*n* = 7)	6.71 ± 1.80		
	30 d	ALRG (*n* = 7)	11.00 ± 1.00	MD: 1.71 (−0.03, 3.46)	0.053
		LRG (*n* = 7)	9.29 ± 1.80		
	60 d	ALRG (*n* = 7)	13.00 ± 1.00	MD: 1.71 (−0.11, 3.54)	0.061
		LRG (*n* = 7)	11.29 ± 1.25		

Legend: Data are presented as mean ± standard deviation. Effect sizes are reported as Mean Difference (MD) with 95% confidence interval derived from paired t-test for parametric data, or as Median Difference with 95% bootstrap confidence interval for non-parametric data. ‡ *p* value not reported for the Rusbridge Grade 2 subgroup due to small sample size, which precluded meaningful statistical testing. N/A Not applicable; a valid statistical test could not be performed due to complete separation or lack of variability in the data after matching.

**Table 5:** Comparison of Analgesic Utilization and Treatment Duration Between ALRG and LRG After PSM, Stratified by Rusbridge Grade and overall.

Rusbridge Grade	Drug Class	ALRG	LRG	Effect Size (95% CI)	*p*
Users					
2	Opioids	1 (33.33%)	2 (66.67%)	‡	‡
(ALRG *n* = 3, LRG *n* = 3)	NSAIDs	3 (100%)	3 (100%)	‡	‡
	Ca^2+^ Modulators	2 (66.67%)	2 (66.67%)	‡	‡
3	Opioids	8 (80%)	9 (90%)	RD: −10.00% (−41.00%, 21.00%)	1.000
(ALRG *n* = 10, LRG *n* = 10)	NSAIDs	10 (100%)	10 (100%)	RD: 0.00% (0.00%, 0.00%)	N/A
	Ca^2+^ Modulators	8 (80%)	8 (80%)	RD: 0.00% (−35.06%, 35.06%)	1.000
4	Opioids	5 (71.43%)	6 (85.71%)	RD: −14.29% (−56.61%, 28.03%)	1.000
(ALRG *n* = 7, LRG *n* = 7)	NSAIDs	7 (100%)	7 (100%)	RD: 0.00% (0.00%, 0.00%)	N/A
	Ca^2+^ Modulators	6 (85.71%)	6 (85.71%)	RD: 0.00% (−36.67%, 36.67%)	1.000
Overall	Opioids	14 (70%)	17 (85%)	RD: −15.00% (−40.50%, 10.50%)	0.451
(ALRG *n* = 20, LRG *n* = 20)	NSAIDs	20 (100%)	20 (100%)	RD: 0.00% (0.00%, 0.00%)	N/A
	Ca^2+^ Modulators	16 (80%)	16 (80%)	RD: 0.00% (−24.80%, 24.80%)	1.000
Duration					
2	Opioids	0 (0–5)	1 (0.5–1.5)	‡	‡
(ALRG *n* = 3, LRG *n* = 3)	NSAIDs	5 (5–5)	7 (6.5–7.5)	‡	‡
	Ca^2+^ Modulators	7 (7–8.5)	14 (7–14)	‡	‡
3	Opioids	0.80 ± 0.42	3.00 ± 1.56	MD: −2.20 (−3.40, −0.99)	0.003
(ALRG *n* = 10, LRG *n* = 10)	NSAIDs	6.30 ± 0.82	6.90 ± 1.37	MD: −6.00 (−1.82, 0.62)	0.297
	Ca^2+^ Modulators	13.50 ± 5.62	13.90 ± 7.88	MD: −4.00 (−6.75, 5.95)	0.890
4	Opioids	1.00 ± 0.82	2.71 ± 1.80	MD: −1.71 (−3.83, 0.40)	0.095
(ALRG *n* = 7, LRG *n* = 7)	NSAIDs	6.86 ± 1.35	6.57 ± 1.61	MD: 0.29 (−1.46, 2.03)	0.703
	Ca^2+^ Modulators	13.86 ± 5.90	16.57 ± 8.18	MD: −2.71 (−12.87, 7.44)	0.537
Overall	Opioids	1 (0–1)	2.60 ± 1.67	MD: 0.20 (0.10, 0.31)	0.001
(ALRG *n* = 20, LRG *n* = 20)	NSAIDs	6 (5.25–7)	6.80 ± 1.36	MD:0.39 (0.28, 0.52)	0.236
2	Ca^2+^ Modulators	13.5 (7–17.75)	15 (14–19.75)	MD: 0.42 (0.30, 0.54)	0.363

Legend: Data are presented as count (%) and as median (interquartile range) or mean ± standard deviation, as appropriate. Effect sizes are reported as risk difference (RD) with 95% confidence interval for categorical variables. For continuous variables, effect sizes are reported as mean difference (MD) with 95% confidence interval. ‡ *p* value not reported for the Rusbridge Grade 2 subgroup due to small sample size, which precluded meaningful statistical testing. N/A Not applicable; a valid statistical test could not be performed due to complete separation or lack of variability in the data after matching.

**Table 6:** Comparison of Long-term prognosis Between ALRG and LRG Groups in Canines After PSM, Stratified by Rusbridge Grade and overall.

Rusbridge Grade	Efficacy Category	ALRG	LRG	*p*
2	Complete success	3 (100%)	3 (100%)	‡
(ALRG *n* = 3, LRG *n* = 3)	Partial success	0 (0%)	0 (0%)	
	Minimal improvement	0 (0%)	0 (0%)	
3	Complete success	9 (90%)	5 (50%)	N/A
(ALRG *n* = 10, LRG *n* = 10)	Partial success	1 (10%)	3 (30%)	
	Minimal improvement	0 (0%)	2 (20%)	
4	Complete success	4 (57.14%)	2 (28.57%)	N/A
(ALRG *n* = 7, LRG *n* = 7)	Partial success	3 (42.86%)	2 (28.57%)	
	Minimal improvement	0 (0%)	3 (42.86%)	
Overall	Complete success	16 (80%)	10 (50%)	0.048
(ALRG *n* = 20, LRG *n* = 20)	Partial success	4 (20%)	5 (25%)	
	Minimal improvement	0 (0%)	5 (25%)	

Legend: Data are presented as count (%). ‡ *p* value not reported for the Rusbridge Grade 2 subgroup due to small sample size, which precluded meaningful statistical testing. N/A Not applicable; a valid statistical test could not be performed due to complete separation or lack of variability in the data after matching.

## Data Availability

All data are contained in the manuscript.

## Abbreviations

The following abbreviations are used in this manuscript:

IVDH	intervertebral disc herniation
VSD	ventral slot decompression
AM	acupuncture–moxibustion
LT	locomotor training
MA	manual acupuncture
EA	electroacupuncture
ALRG	Acupuncture–Moxibustion and Locomotor Rehabilitation Group
LRG	Locomotor Rehabilitation Group
PSM	propensity score matching
TCVM	Traditional Chinese Veterinary Medicine
UWTM	underwater treadmill
PROM	passive range of motion

## Appendix A

**Table A1:** Therapeutic acupoints and their anatomical localization in canines.

Acupoint Name	Localization
Jing-jiaji (cervical jiaji)	0.5–1.5 cm lateral to cervical spinous processes (adjacent to lesion segments)
BL-11 (Da zhu)	1.5 cm lateral to T1 spinous process
LU-7 (Lie que)	1–2 cm proximal to medial carpal joint
LI-4 (He gu)	Proximal 1/3 of 2nd-3rd metacarpal space
KID-3 (Tai xi)	Depression between medial malleolus and Achilles tendon
BL-20 (Pi shu)	1.5–2 cm lateral to T11 spinous process
BL-23 (Shen shu)	1.5–2 cm lateral to L2 spinous process
BL-18 (Gan shu)	1.5–2 cm lateral to T9 spinous process
ST-36 (Zu san li)	0.5 cm lateral to tibial crest, distal to knee
GB-20 (Feng chi)	Depression between occipital crest and atlas wing
LIV-3 (Tai chong)	Proximal depression of 2nd-3rd metatarsal space

**Table A2:** Detailed Breed Distribution Before and After Matching, Stratified by Rusbridge Grade (Counts Only).

	Rusbridge Grade 2		Rusbridge Grade 3		Rusbridge Grade 4	
Breed	Pre-Match	Post-Match	Pre-Match	Post-Match	Pre-Match	Post-Match
	ALRG/LRG	ALRG/LRG	ALRG/LRG	ALRG/LRG	ALRG/LRG	ALRG/LRG
	(n = 10)/(n = 9)	(n = 3)/(n = 3)	(n = 17)/(n = 10)	(n = 10)/(n = 10)	(n = 20)/(n = 13)	(n = 7)/(n = 7)
Beagle	1/0	0/0	1/0	0/0	1/0	0/0
Bichon Frisé	0/2	0/1	3/0	2/0	2/3	1/0
Border Collie	0/0	0/0	0/0	0/0	0/0	0/0
Chihuahua	2/2	0/1	0/0	0/0	0/0	0/0
Chinese Rural Dog	0/0	0/0	3/0	1/0	1/2	1/0
Cocker Spaniel	0/0	0/0	0/0	0/0	0/1	0/0
Dachshund	1/0	1/0	0/0	0/0	0/1	0/0
French Bulldog	0/1	0/0	0/1	0/1	1/1	0/1
Golden Retriever	0/1	0/0	0/0	0/0	0/1	0/1
Labrador Retriever	0/0	0/0	1/0	0/0	0/0	0/0
Maltese	0/0	0/0	0/1	0/1	2/0	1/0
Miniature Pinscher	0/0	0/0	0/0	0/0	1/0	0/0
Mixed-breed Dog	1/0	0/0	0/1	0/1	5/2	2/2
Papillon	0/0	0/0	1/0	1/0	0/0	0/0
Pekingese	0/0	0/0	0/1	0/1	0/0	0/0
Pembroke Welsh Corgi	0/0	0/0	0/1	0/1	0/1	0/1
Pomeranian	0/1	0/0	1/1	0/1	0/0	0/0
Poodle	3/1	1/1	5/3	4/3	5/2	1/2
Pug	0/0	0/0	1/0	1/0	0/0	0/0
Schnauzer	1/0	1/0	0/1	0/1	0/1	0/0
Shih Tzu	0/0	0/0	1/0	1/0	0/0	0/0
Tibetan Mastiff	0/1	0/0	0/0	0/0	1/0	0/0
Yorkshire Terrier	1/0	0/0	0/0	0/0	1/0	1/0
